# Supplementary material for: Enhanced Silk Fibroin/Sericin Composite Film: Preparation, Mechanical Properties and Mineralization Activity
Source: Polymers (Basel). 2022 Jun 17;14(12):2466. doi: 10.3390/polym14122466 (PMC9227074; doi:10.3390/polym14122466)
Supplement: Supplementary file 1 [file polymers-14-02466-s001.zip › polymers-1761365-supplementary.pdf]

Table S1. Molecular conformation content of SF/SS composite films (%).

	Sample	β-sheet	Random coil	α-helix	β-turn
No treatment	SF	44.48	23.12	9.53	22.86
	SF/SS_4	47.34	21.23	8.94	22.49
	SF/SS_3	44.94	25.65	9.28	20.12
	SF/SS_1	44.73	22.49	9.53	23.26
Ethanol treatment	SF	52.11	19.54	7.25	21.09
	SF/SS_4	68.98	9.29	1.40	20.33
	SF/SS_3	52.15	9.88	11.11	26.87
	SF/SS_1	48.93	11.93	17.56	21.58

Table S2. Tensile properties of SF/SS composite films by ethanol treatment.

	SF	SF/SS_4	SF/SS_3	SF/SS_1
Breaking elongation (%)	56.34±16.31	98.39±11.69	133.75±27.75	135.68±22.94
Breaking strength (MPa)	5.36±0.53	6.69±0.59	8.83±1.25	7.71±1.35
Young's modulus (Mpa)	56.48±12.43	51.69±9.64	53.95±4.06	35.94±2.91

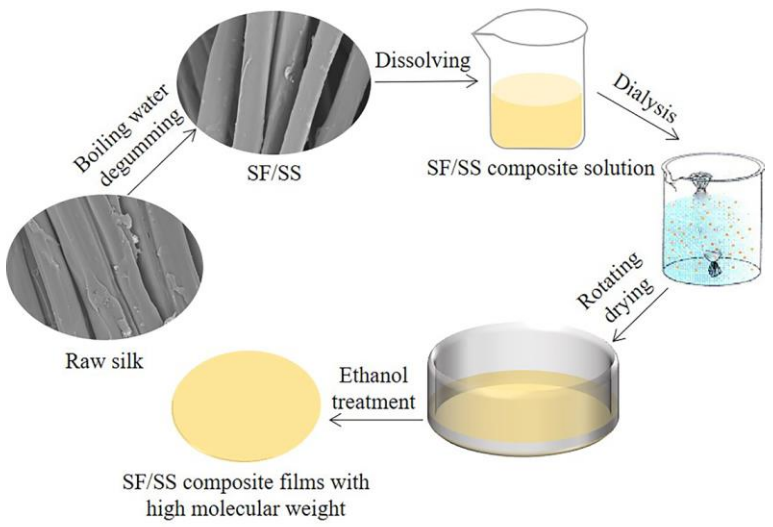

Figure S1. The illustration representing preparation of SF/SS composite films.

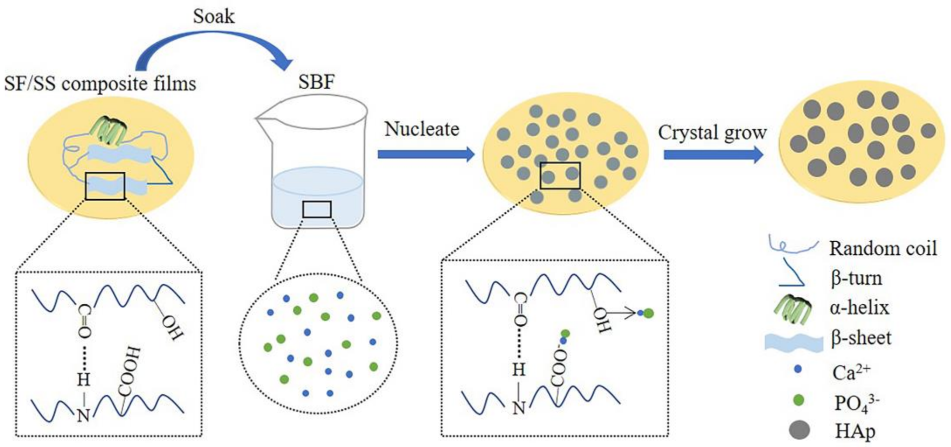

Figure S2. The Schematic diagram of mineralization on SF/SS composite films.

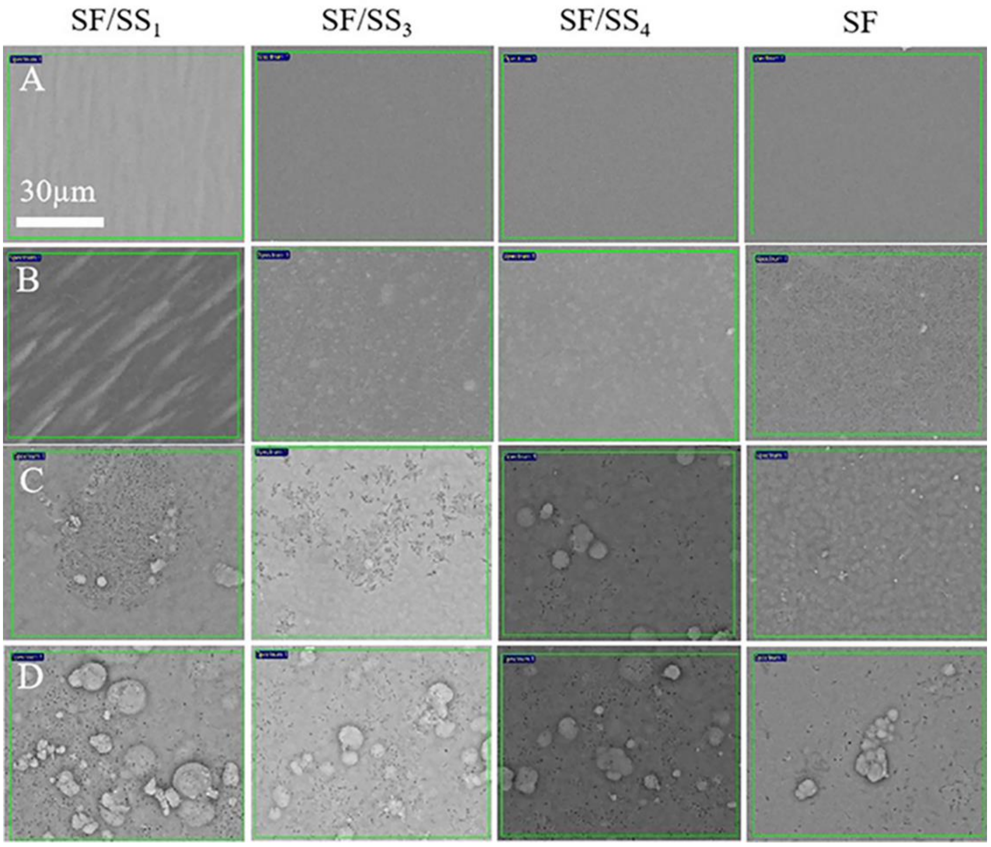

Figure S3. The distribution of growing crystals after mineralization. A: Unmineralized; B: 1 d; C: 3 d; D: 5 d.

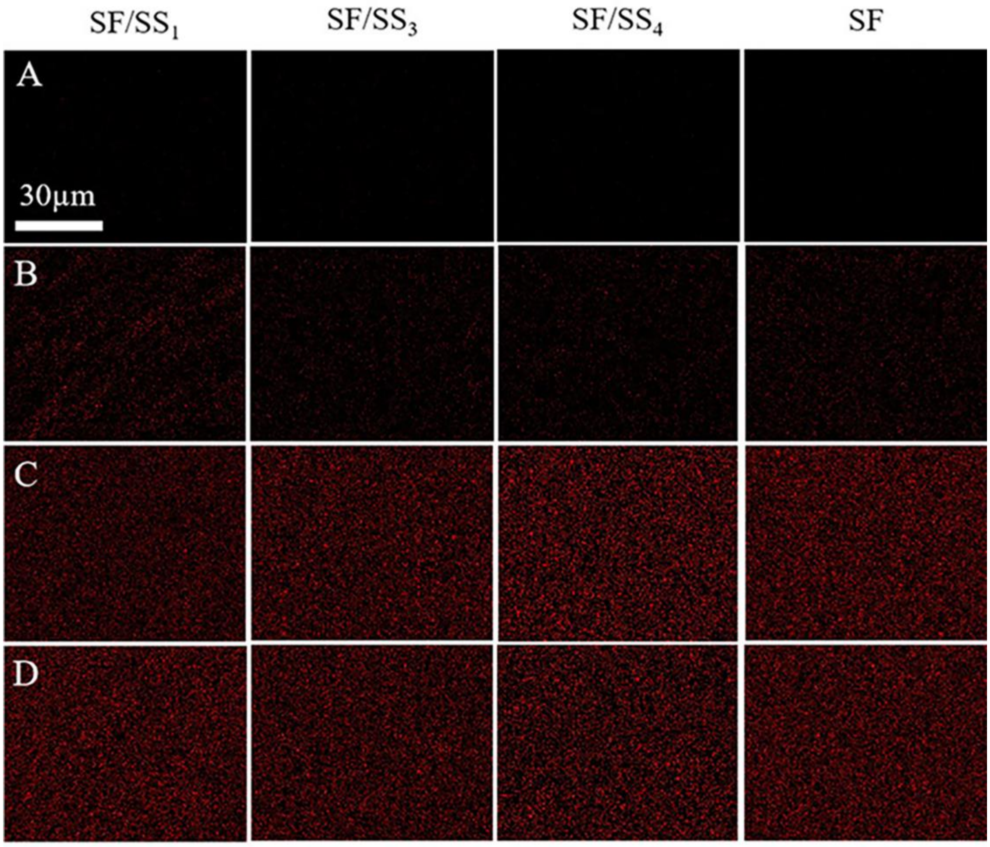

Figure S4. The distribution of calcium element after mineralization. A: Unmineralized; B: 1 d; C: 3 d; D: 5 d.

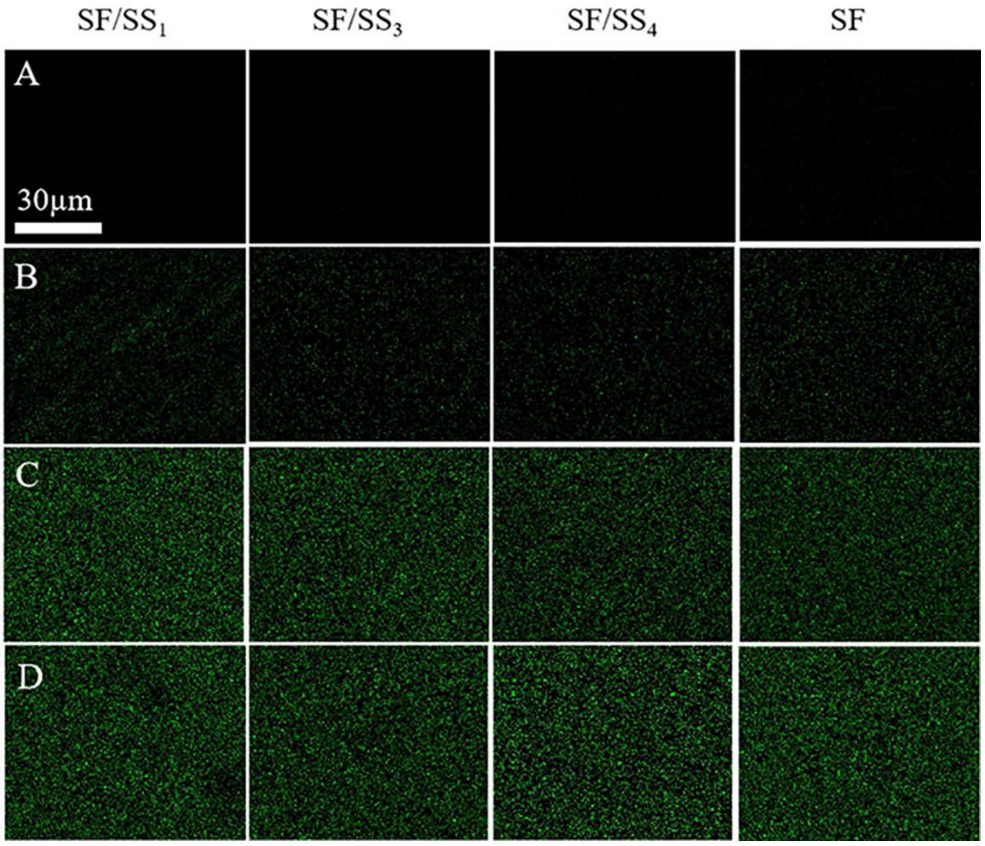

Figure S5. The distribution of phosphorus element after mineralization. A: Unmineralized; B: 1 d; C: 3 d; D: 5 d.
